# Prevalence of respiratory viruses among children hospitalized from respiratory infections in Shenzhen, China

**DOI:** 10.1186/s12985-016-0493-7

**Published:** 2016-03-08

**Authors:** Heping Wang, Yuejie Zheng, Jikui Deng, Wenjian Wang, Ping Liu, Fanghua Yang, Hanfang Jiang

**Affiliations:** Department of Respiratory Diseases, Shenzhen Children’s Hospital, 7019 Yitian Road, Futian District, Shenzhen, 518026 China

**Keywords:** Prevalence, Acute respiratory virus infection, Hospitalized children, Shenzhen

## Abstract

**Background:**

The prevalence of local dominant viral etiologies is important for clinical management and prevention of common viral respiratory tract infections. Unfortunately, there is limited large-scale data about common viral respiratory infection in south China. To survey dominant viral etiology and seasonality of acute respiratory infections in hospitalized children, a 4-year consecutive study was conducted in Shenzhen, China.

**Methods:**

Nasopharyngeal swab specimens were obtained from 30,443 hospitalized children younger than 14 years with respiratory tract diseases in Shenzhen Children’s Hospital from January 2012 to December 2015. Nasopharyngeal swabs were routinely examined by direct immunofluorescence assay to detect respiratory agents including seven respiratory viruses. Data were analyzed to describe the frequency and seasonality.

**Results:**

Of the 30,443 children enrolled in the study, 4428 (14.55 %) were positive for at least one viral pathogen, among whom 4110 (92.82 %) were ≤3 years of age. The predominant viruses were respiratory syncytial virus (RSV, 68.11 %), adenovirus (ADV, 16.01 %) and parainfluenza virus 3 (PIV-3, 11.0 %). The common respiratory viruses detected peaked in the spring (17.69 %), and were minimal in autumn (9.73 %), but PIVs detection peaked in November. The common virus detection rate in male subjects (15.40 %) was significantly higher than in female subjects (13.02 %). PIVs detection rates were complementary with RSV in autumn in each year.

**Conclusions:**

This study demonstrated common respiratory viruses were the major cause of hospitalized acute respiratory infection (ARI) in children in Shenzhen, China. RSV was the most common detected infection, while ADV was the predominant pathogen in hospitalized children. These findings provide a better understanding of virus distribution among children of different ages, infection stratification by gender, and seasonality, all of which will contribute to modification of therapeutic approaches and development of effective prevention strategies for each respiratory virus infection during peak seasons.

## Background

Acute respiratory infections (ARIs) are a persistent and pervasive public health problem in both developed and developing countries, causing nearly four million deaths per year, a rate of more than 60 deaths/100,000 population [1]. These rates are especially higher in developing countries including China. Many pathogens can cause ARIs, and viruses have been considered the main pathogens [2, 3]. The most frequently reported viruses include respiratory syncytial virus (RSV), *Influenza A* and *B* viruses (IAV, IBV), parainfluenza viruses (PIVs), adenovirus (ADV), and human rhinovirus (HRV), which are responsible for most episodes of ARIs in children.

At present, there are no approved vaccines or medications available for most of these respiratory viruses [2]. A better understanding of the common viruses of hospitalized ARIs in children is critical for the successful implementation of prevention, control, and treatment strategies. The prevalence of each respiratory virus varies from country to country, and may be due to differences in viral strains, seasonal timing, and geographic areas. China is a large country with varying climate characteristics in different regions. Although some studies on the epidemiology of ARIs have recently been reported in big cities such as Beijing, Shanghai, and Hong Kong [4–6], the epidemic characteristics of common viruses in ARIs are still not well established in other parts of China, especially in smaller cities and rural areas.

Shenzhen is a large migratory city of China with high population density and population mobility [7]. Shenzhen is located in southern China, immediately north of Hong Kong, with a typical subtropical monsoon climate. We undertook a public health service initiative to understand pediatric ARI within Shenzhen. Therefore, this study, conducted at the Shenzhen Children’s Hospital, recruited the majority of hospitalized children. We explored the prevalence of common viruses of ARIs of hospitalized children in Shenzhen, which could assist in the prevention, control, and treatment of ARIs.

## Results

### Patient characteristics and clinical diagnosis

A total of 30,443 consecutive hospitalized children with respiratory tract infection were enrolled from Jan 2012 to December 2015. The ages of the children ranged from 8 days to 14 years old. The vast majority of patients (82.97 %) were ≤3 years old. We divided the patients into four groups: (i) infant group (newborn ~1 year old), 19,412 cases; (ii) toddler group (>1 ~ 3 years old), 5836 cases; (iii) pre-school group (>3 ~ 6 years old), 3627 cases; and (iv) school children group (7 ~ 14 years old), 1568 cases. The subjects included 19,462 males (63.93 %) and 10,981 females (36.07 %) (sex ratio, 1.77:1). According to the seasons, all the patients were divided into four groups: (i) spring group (March, April and May), 7878 cases; (ii) summer group (June, July and August), 8031 cases; (iii) autumn group (September, October and November), 8018 cases; and (iv) winter group (January, February and December), 6516 cases.

### Overall detection percentage of seven viruses

Of the total 30,443 specimens, 4428 (14.55 %) tested positive for at least one of the seven viruses. The overall detection rates in each year between 2012 and 2015 were 17.16 %, 15.19 %, 13.13 %, and 14.53 %, respectively. The most predominant virus was RSV, with a detection rate of 68.11 % (3016 of 4428). The viruses ranking second to fifth were ADV, PIVs, IAV, and IBV, with detection rates of 16.01 %, 12.22 %, 2.73 %, and 0.93 %, respectively (Table 1).

**Table 1:** Detection percentages of 7 viruses

Viruses	Number positive	Percentage (%)
Respiratory syncytial virus (RSV)	2971	68.36 %
Adenovirus (ADV)	683	15.72 %
Parainfluenza 1, 2 and 3 (PIVs)	530	12.20 %
Influenza A (IAV)	121	2.78 %
Influenza B (IBV)	41	0.94 %

### Detection percentages of viruses from different age groups and gender distribution

All of the ARI patients were grouped into four age groups with different detection rates of viral infections (Table 2). The total detection rate decreased with rising age of the enrolled children, 17.58 % in infants, 11.96 % in toddlers, 7.0 % in preschoolers, and 4.08 % in school aged children. The specific detection rate of RSV and PIVs also followed a similar pattern as the total rate, wherein higher rates were detected in younger children. However, the detection rate of IBV increased with age, while ADV and IAV were detected predominantly in toddlers and the least in infants.

**Table 2:** Detection percentages of viruses from different age groups

Viruses	Infants (%) *N* =19,412	Toddlers (%) *N* =5836	Preschooler (%) *N* =3627	School children (%) *N* =1568	*X* ^2^	*P*
Respiratory syncytial virus (RSV)	2599 (13.39 %)	344 (5.89 %)	65 (1.79 %)	9 (0.57 %)	784.351	0.001
Adenovirus (ADV)	307 (1.58 %)	228 (3.91 %)	139(3.83 %)	35 (2.23 %)	140.353	0.001
Parainfluenza 1, 2 and 3 (PIVs)	440 (2.27 %)	74 (1.28 %)	21 (0.58 %)	5 (0.32 %)	79.491	0.001
Influenza A (IAV)	52 (0.27 %)	45 (0.77 %)	17 (0.47 %)	7 (0.45 %)	29.649	0.001
Influenza B (IBV)	14(0.07 %)	7(0.12 %)	12(0.33 %)	8(0.51 %)	33.442	0.001
Total	3364(17.79 %)	680(12.01 %)	240(6.90 %)	62(4.15 %)	477.770	0.001

The predominant viruses among different age groups varied. In infants (≤1 years), RSV (13.39 %, 2599 of 19,412) was the most prevalent virus followed by PIVs (2.27 %, 440 of 19,412), ADV (1.58 %, 307 of 19,412), IAV (0.27 %, 52 of 19,412) and IBV (0.07 %, 14 of 19,412). In toddlers and preschoolers, ADV (3.91 %, 228 of 5836) and RSV (5.89 %, 344 of 5836) had the highest incidence, followed by PIVs (1.28 %, 74 of 5836), IAV (0.77 %, 45 of 5836), and IBV (0.12 %, 7 of 5836). In school children, ADV (3.83 %, 139 of 3627) was the most predominant virus. All the detection rates of different virus were found to be associated with age, with *p* values of <0.001 in all viruses (Table 2). The detection rates of common viruses in male and female were 15.40 % and 13.02 %, respectively. The detection rate of males was significantly higher than female subjects (*X*2 = 32.351, *p* < 0.001).

### Detection percentages of viruses from different seasons

**Fig. 1:**
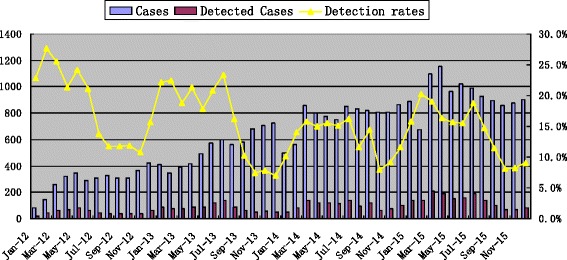
Monthly distribution and detection rates of acute respiratory tract infection cases in 30,443 inpatient children, January 2012–December 2015

In general, common respiratory viruses were detected more often in spring, summer and winter than autumn (*X*2 = 237.999, *p* < 0.001), and obvious seasonal peaks were observed during those months with peak strength varying from one year to another (Fig. 1). Among the four major viruses, the detection rates of RSV, PIVs, ADV, and IAV were found to be associated with seasons. Considering all examined viruses, IAV and ADV were mostly detected in winter; PIVs mostly in autumn, especially in November; while RSV was detected primarily during the spring and summer. In autumn, except for the PIVs, the detection rates of other viruses were the lowest in four seasons (Table 3).

**Table 3:** Detection percentages of viruses from different seasons

Viruses	Spring (%) *N* =7878	Summer (%) *N* =8037	Autumn (%) *N* =8018	Winter (%) *N* =6516	*X* ^2^	*P*
Respiratory syncytial virus (RSV)	1028 (13.05 %)	1004 (12.50 %)	421 (5.25 %)	563 (8.64 %)	340.028	0.001
Adenovirus (ADV)	158 (2.13 %)	200 (2.49 %)	144 (1.80 %)	197 (3.02 %)	25.115	0.001
Parainfluenza 1, 2 and 3 (PIVs)	147 (1.87 %)	83 (1.03 %)	196 (2.45 %)	115 (1.76 %)	45.959	0.001
Influenza A (IAV)	40 (0.51 %)	32 (.040 %)	16 (0.20 %)	33 (0.51 %)	14.932	0.002
Influenza B (IBV)	11 (0.14 %)	3 (0.04 %)	3 (0.04 %)	24 (0.37 %)	45.681	0.001
Total	1394 (17.69 %)	1322 (16.46 %)	780 (9.73 %)	850 (15.14 %)	234.999	0.001

**Fig. 2:**
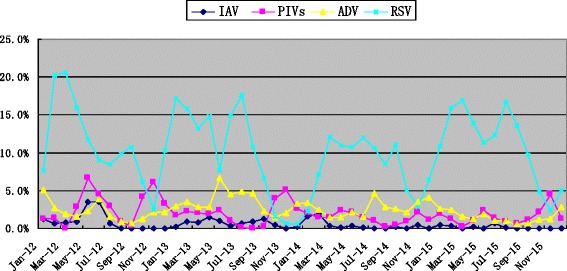
Monthly distribution of single virus detected in 30,443 inpatient children, January 2012–December 2015

The detection rates of RSV in March and September and of ADV/PIVs in May and November were higher than in other months. RSV was detected almost throughout the whole year, with its detection rate of RSV in November lower than in other months varying from the first year to the next year. Our investigation did not find regular seasonality in IAV detection, a mild peak of detection rate was observed from spring to summer in 2012–2015 (Fig. 2).

## Discussion

There have been few long-term prospective studies conducted for common viral etiologies of ARIs among hospitalized children. RSV, ADV, PIVs, and IAV are the most important etiological viruses of ARIs in infants and young children in Shenzhen, which have been detected previously by RT-PCR or PCR methods [7]. However, those data were based on small cases, therefore, we conducted a large study from 2012 to 2015 to assess the regional common virus infection pattern in children in Shenzhen, China. The common respiratory viruses including RSV, ADV, PIVs, and INF were detected by DFA, and then the infection frequency, age distribution, and seasonality of viral respiratory infections were analyzed.

In this study, we evaluated the overall prevalence of the most frequent respiratory viruses based on prospective analysis of four consecutive years’ data from hospitalized children with ARIs. The overall detection rate for one or two of the seven respiratory viruses by DFA was 14.55 % (4428/30,443) in hospitalized children with ARIs in Shenzhen, China, and the detection rate was relatively stable in each year. The overall detection rate in our study was lower than that reported recently in China [8–12]. The overall positive rate reported varied from 16.45 to 62.6 % depending on different areas, study design, and detection methods. The overall rate of detection in our study was lower than that reported recently in Shenzhen (Deng J et al., 62.6 % and He Y et al., 48.0 %), Gansu (37.6 %), Shandong (35.75 %) and 22 provinces of China (36.6 %), likely as a result of employing PCR methodology and detection of additional virus strains in these studies. Nonetheless, in analyzing the same samples using DFA, the finding was lower than Shenzhen (16.45 %) and Chongqing (48.26 %) mainly because of variation in sample collection time or geographical differences.

Our study utilized DFA instead of PCR method, since DFA can independently and simultaneously detect a variety of different viruses without interference with each other. Based on previous research, although PCR is more sensitive for virus detection, DFA offers a reliable point-of-care alternative detection method especially during early phase of illness [13]. The sensitivity of DFA in comparison to rt-RT-PCR was highest (86 %) during the first 3 days of symptoms onset and decreased gradually until it reached 65 % after the first week.

The age distribution in this hospital-based study indicated that infants were more likely to be infected by RSV and PIVs, toddlers and preschoolers with ADV and RSV, and school children with ADV. However, IAV was not predominant in any group. Previous studies indicated that IAV was the most prevalent virus in children with viral respiratory infection in China [8–10]. However, those studies were conducted during IAV outbreak periods, which unduly influenced viral distribution patterns. A significant gender difference was revealed in our study. The detection rate in male patients was higher than in female patients, which was different from many small sample size studies and coincided with the large size study in Chongqing, China [12].

The difference of seasonal virus detection may be related to a region’s climate and demographic factors. The detection rate was lowest in the autumn and highest in the spring in this study in Shenzhen, China. This was different from previous studies which showed that highest detection rates were in the winter or summer [14, 15]. The seasonal characteristics of RSV, PIVs, and ADV may be related to a region’s climate and demographic factors. Our results indicated that the detection rate of PIVs was highest in autumn, while the rate of RSV was lowest in the same season. The detection rate of ADV showed two peaks in each year in our study, highest in winter and then summer. The seasonal detection characteristics of RSV, PIVs, and ADV reported here were different from other studies, such as reports from Shandong, China, Japan and the United States [15–18].

## Conclusions

The findings presented in this study provide a better understanding of virus distribution among different pediatric ages, genders, and seasons in Shenzhen, China, which may contribute to modification of therapeutic approaches and development of effective prevention strategies for each respiratory virus infection during its specific peak season(s). Our study demonstrates that respiratory adenovirus infection is an important cause of hospitalizations in children in Shenzhen, China. Although PCR is more sensitive for virus detection, DFA offers a reliable point-of-care alternative detection method, especially during the early phase of illness.

## Methods

### Patients and specimens

A consecutive 4-year prospective study from January 2012 to December 2015 was conducted at the Shenzhen Children’s Hospital. Selected patients with ARIs admitted to the pediatric wards were enrolled. The inclusion criteria were as follows: less than 14 years old, included one or more respiratory symptoms (cough, sore throat, combined with a body temperature above 37.5 °C). The other information including symptoms, clinical diagnosis and demographic characteristic were recorded in case report forms. Nasopharyngeal swabs were obtained by trained personnel following standard operating procedures within 24 h after admission. The study protocol was approved by the Ethical Committee of Shenzhen Children’s Hospital. Written informed consent was obtained from all participants’ guardians.

### Detection of common respiratory viruses

The specimens were transported immediately to the laboratory by sterile viral transport media, the epithelial cell suspension in phosphate buffered saline was prepared for each sample and air-dried on multi-well Teflon slides, then tested for seven respiratory viruses (including respiratory syncytial virus, adenovirus, influenza virus types A/B and parainfluenza virus types 1, 2, and 3) with direct immunofluorescence assay kits (Diagnostic Hybrids, Inc. USA) by trained personnel according to the manufacturer’s protocol [6].

### Statistical analysis

Statistical analyses were conducted using SPSS 17 (SPSS Inc. Chicago, IL, USA). For comparison of categorical data, chi-square or Fisher’s exact test was used. *P*-value <0.05 was considered to be statistically significant.
